# Method of treating alcohol dependence complicated by amnestic disorders

**DOI:** 10.1192/j.eurpsy.2021.1502

**Published:** 2021-08-13

**Authors:** I. Sosin, Y. Babenko, O. Honcharova, G. Mysko, O. Sergienko, I. Lisova

**Affiliations:** 1 Narcology Department, Kharkiv, Kharkiv Medical Academy of Postgraduate Education, Kharkiv, Ukraine; 2 Narcology Department, Kharkiv Medical Academy of Postgraduate Education, Kharkiv, Ukraine

**Keywords:** pharmacotherapy, Alcohol addiction, Amnestic disorders

## Abstract

**Introduction:**

Amnesia and palimpsests occurring and recurring in alcohol addicts due to alcohol intoxication (Ebrietas alcoholica) are accompanied by hazardous memory failures, gradual mental degradation and psychoorganic syndrome, which evidences urgent clinical, therapeutic and therapeutic issue in addictology, psychiatry, forensic medicine, sociology, medical psychology, etc. At EPA initiative (2019), research interest in non-invasive brain stimulation tools and methods for such populations was activated.

**Objectives:**

Development of a patentable method of treatment in addictology using pyracetam and nicotinic acid transcerebral electrophoresis (TCE).

**Methods:**

Valid clinical-diagnostic, laboratory, biochemical, electrophysiological, psychological (scaling, testing), statistical methods for identification of alcohol dependence complicated by amnestic disorders.

**Results:**

The method of treatment of alcohol dependence complicated by amnestic disorders (Patent 141785 UA) provides complex pharmacological and drug-free therapy. Antiamnesic drugs are administered by TCE bilaterally; pyracetam 20% solution to the left orbit through active negative electrodes, and nicotinic acid 0.1% solution to the right orbit (positive electrode in the occipital fossa), current of 2-4 mA, 20-30 minutes exposure. The procedure was performed daily with a TCE device, for a 10-day course of treatment along with psychotherapeutic potentiation. TCE provides the ionic implementation of pharmacological agents in the brain and their physiological electrical stimulation.

**Conclusions:**

In a representative clinical trial, using statistical methods and generated bank of patient-specific observations, significant potentiating effects of combined drug-free, non-invasive transcerebral electrical stimulation and electrophoretic implementation of pyracetam and nicotinic acid were demonstrated.

